# Structural Elements of the Biomechanical System of Soft Tissue

**DOI:** 10.7759/cureus.7895

**Published:** 2020-04-30

**Authors:** Harm Jaap Smit, Phil Strong

**Affiliations:** 1 Molecular Biology, Independent Researcher, Amersfoort, NLD; 2 Independent Researcher, Oldbury-on-Severn, GBR

**Keywords:** force, tissue, adaption, tissue damage, tissue viability, wounds, biotensegrity, biomechanical systems, prestress, extracellular matrix

## Abstract

In living organisms, forces are constantly generated and transmitted throughout tissue. Such forces are generated through interaction with the environment and as a result of the body’s endogenous movement. If these internally or externally originating forces exceed the ability of tissues to cope with the applied forces, (i.e. “tissue thresholds”), they will cause force-related tissue harm. However, biotensegrity systems act to prevent these forces from causing structural damage to cells and tissues. The mechanism and structure of soft tissues that enable them to maintain their integrity and prevent damage under constantly changing forces is still not fully understood. The current anatomical and physical knowledge is insufficient to assess and predict how, why, where, and when to expect force-related tissue harm.

When including the concept of tensegrity and the related principles of the hierarchical organisation of the elements of the subcellular tensional homeostatic structure into current biomechanical concepts, it increases our understanding of the events in force handling in relation to the onset of force-related tissue harm: Reducing incident forces in tissue to a level that is not harmful to the involved structures is achieved by dissipation, transduction and transferring the force in multiple dimensions. To enable this, the biomechanical systems must function in a continuous and consistent way from the cellular level to the entire body to prevent local peak forces from causing harm. In this article, we explore the biomechanical system with a focus on biotensegrity concepts across several organisational levels, describing in detail how it may function and reflecting on how this might be applied to patient management.

## Introduction and background

Within the body tissues, simple activities, for example, moving a limb or even breathing, require physiological structures that permit sliding and deformation of tissue(s). The sliding and deformation of tissue(s) generate forces that, at any point, should not exceed the amount of force a cell or tissue can handle (the “tissue threshold”) or damage may occur.

The necessary sliding or “relative movement” as identified above between two subjects may challenge both the structural integrity and the function of the intra- and extra-cellular transport systems [1].

There are also challenges in the different mechanical properties of tissue involved in handling forces., Forces on body parts consisting of tissues with very different mechanical properties (hard or soft tissue) may cause a deformity in a non-uniform way across the differing tissue types unless there is a system in place that handles the forces in relation to the involved tissue. This also applies to the tissues involved in the formation of decubitus.

Another interesting aspect is that tissue damage caused by pressure and shear forces is not exhibited uniformly across individuals. It is a common observation to see dramatically different outcomes in force-related tissue damage in clinically comparable or compromised patients.

Biomechanical aspects in whole living organisms are usually represented as a system of bones, muscles and tendons in a lever system [2]. However, when focussing down on soft tissue specifically, these macro-elements are absent yet soft tissues are still able to maintain their structural integrity.

As animals and humans move around, the inner body itself is also in constant movement: As well as the movements dependent on skeletal muscles, the cardiovascular and respiratory systems also depend on movement for function. As a result, cells and tissues are continuously subjected to forces and the resulting perpetual deformations. This, in turn, requires continuous rearrangement of internal structures and energy expenditure. The body aims to preserve energy and, therefore, to prevent unnecessary deformation. It achieves this by having an internal system that provides cells and tissues with just enough rigidity to withstand force with minimum energy expenditure.

A key element of the cellular strategy for maintaining structural integrity is rigidity. Cellular and tissue rigidity is fine-tuned; it is rigid enough to allow them to maintain its desired shape without interfering with its function. However, if the forces acting on a cell or a tissue become too great, they will deform beyond normal ranges and at a certain point, this can cause harm. As most body parts are occasionally or regularly exposed to damaging forces, all cells and tissues need a system to prevent forces exceeding the tissue threshold described earlier. Usually, this is done by dispersing a force over a larger area by stiffening, stretching or transferring it to mechanically different structures such as skin, bone, tendon and/or adipose tissue.

In summary, to prevent unnecessary harm at any point in the body, the protective system requires two key attributes: firstly, it must function in a continuous fashion from micro to macro (and vice versa) and secondly, it has to be robust; function under all circumstances.

## Review

Forces

The application of a force on a body represents an amount of energy being transferred to that body. The energy will lead to movement, deformation and/or increased shear stress and pressure in and on tissue. In this context, a force and its resultant impact have three key characteristics:

1. The magnitude of a force impacting a structure: a biological structure has a certain capacity for the amount of force it can withstand per surface or volume.

2. The direction of the force: most structures are anisotropic and the magnitude of force a cell or tissue can handle depends on the direction and point of impact of the applied force.

3. The duration of time a force is applied: structures in the body can handle forces up to a certain magnitude for a limited time or need time to adapt to it.

**Figure 1:**
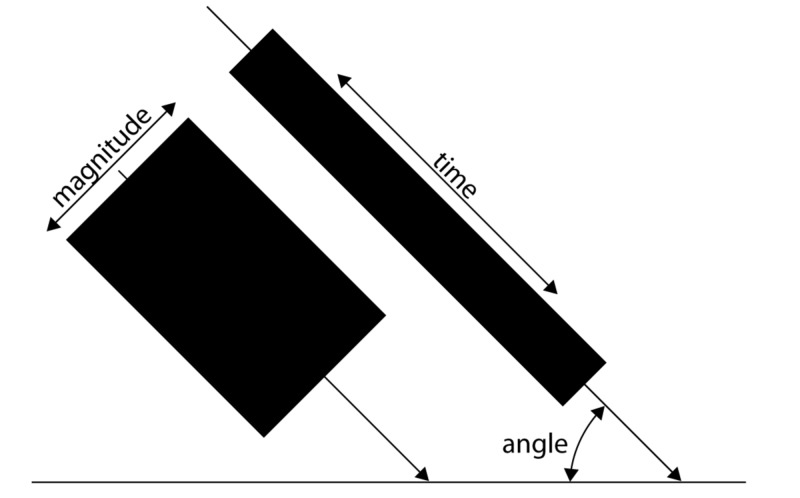
Graphical representation of the key characteristics of a force The surface of the boxes represents the amount of energy a given force can transfer.

Figure 1 shows the three key characteristics.

Applied force may cause stress, damage and other forms of harm, for example, a weightlifter can lift a very large weight but only for a very short period without risk of incurring damage. However, living structures are able to change their structure to adapt allowing them to handle an otherwise damaging force [3]. A typical example of this is the formation of a callus or collagen arrangement which enables cells to handle damaging forces. To adapt in this way, a cell or tissue needs time. Additionally, the body may also adapt to forces by adjusting its behaviour such as repositioning or changing a movement.

Bio-mechanisms for handling forces

The biomechanical system has a role to redistribute an external force while maintaining and safeguarding tensional homeostasis [4]. Tensional homeostasis describes the structural integrity that allows a cell and/or a tissue to maintain its mechanical structure and is required for the correct function of the involved cell or tissue.

**Figure 2:**
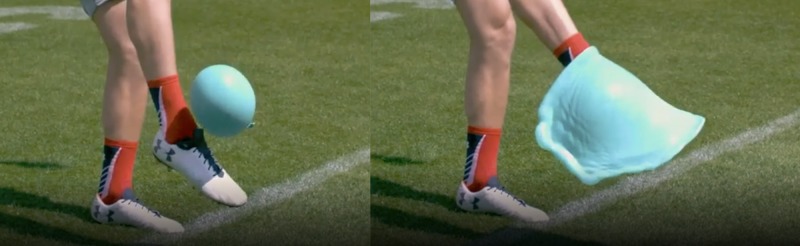
Dissipating force through displacement: before and after

Functional cellular shape can be maintained differently across species and kingdoms: for example, plant cells can maintain shape using turgor (osmotic forces leading to increased internal pressure on an outer layer or cell wall). The problem with a cell with a rigid outer layer is that it cannot change shape or move. In animals, an effective way to achieve rigidity in cells and their aggregate tissues is also by increased cellular pressure on an outer layer or cell wall. This can be achieved through the osmotic properties of the cytoplasm [5]. However, increased osmotic pressure reduces not only malleability but also signalling cascades, which pose problems in human cells and the tissues they are in [1]. Although cellular pressure is part of the tensegrity system, cellular pressure is not the solution to cellular shape and adaptability in its entirety because if cytoplasm was completely liquid in character, the absence of either rigid cell walls or other containing structures would result in increased cellular pressure, forcing the cell into a perfect sphere and, therefore, compromise its ability to achieve the shape needed for its differentiated function [6]. However, from the perspective of response to outside force impact, cellular pressure and its resulting fluid-like behaviour of cells is effective in dissipating force. Figure 2 shows that not only is the force handled by displacing fluid, but it can also be handled by displacing the force itself, like waves in the ocean.

In addition to the compromise in differentiated functional shape, relying on cellular pressure alone for structural integrity would impose an onerous burden of energy usage. To achieve adaptable strength, as well as cellular pressure-based strength, the elements of the tensegrity structural hierarchy must be considered.

The mechanism nature uses for this adaptable structural strength is to apply force to a structure, resulting in rigidity, for example, bending a bow with string. This is called prestress [7]. Technically, this is achieved through stressing or tensioning structures to increase their rigidity.

In 1998, Donald Ingber, inspired by the tensegrity work of the American Architect Buckminster Fuller, showed how a simple mechanical model of cell structure based on tensegrity architecture can help to explain how cell shape, movement and cytoskeletal mechanics are controlled and, as a result, how cells sense and can respond to mechanical forces [6].

The cell and the smallest structures in the body are surrounded and invaginated by the Extra Cellular Matrix (ECM), which act as a tensegrity scaffold for the cell [8].

**Figure 3:**
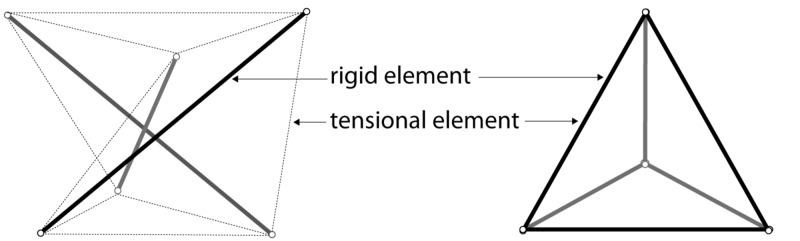
Two types of tensegrity elements

Fascia can neutralise the gravitational force on the body and prevent it from collapsing [9]. Ingber showed how something as soft as a cell or a group of cells can maintain its form in very diverse circumstances and how this integrity is actively used to change shape and/or move. This is called “Tensegrity”. Tensegrity is achieved by placing tension on a rigid element, which increases its strength. Combining tensioned elements allows for a strong yet light structure, as can be seen in a Buckminster Fuller dome. If the tension is delivered at an elastic element, the entire system of elastic and rigid elements becomes a flexible structure, which has an innate “memory” of its original shape. The concept of a tensegrity system is demonstrated in living systems. It allows for a continuum of a tensional homeostatic structure from the cellular nucleus to the entire body [10]. A tensegrity system functions similarly to the spokes, which provide stiffness to a wheel [8]. Likewise, a tensegrity system consists of two key elements, a rigid element and a tensional element, however, in a Buckminster Fuller construction, metal struts function both as a tensional element and a rigid element, as seen in a tetrahedron. Similar structures can be seen in nature (Figure 3).

Stephen Levin referenced the adaptive tensegrity occurrence in living organisms as “biotensegrity” [11]. Biotensegrity is a system that allows for a varying level of rigidity, which is achieved through adaptable elements.

**Figure 4:**
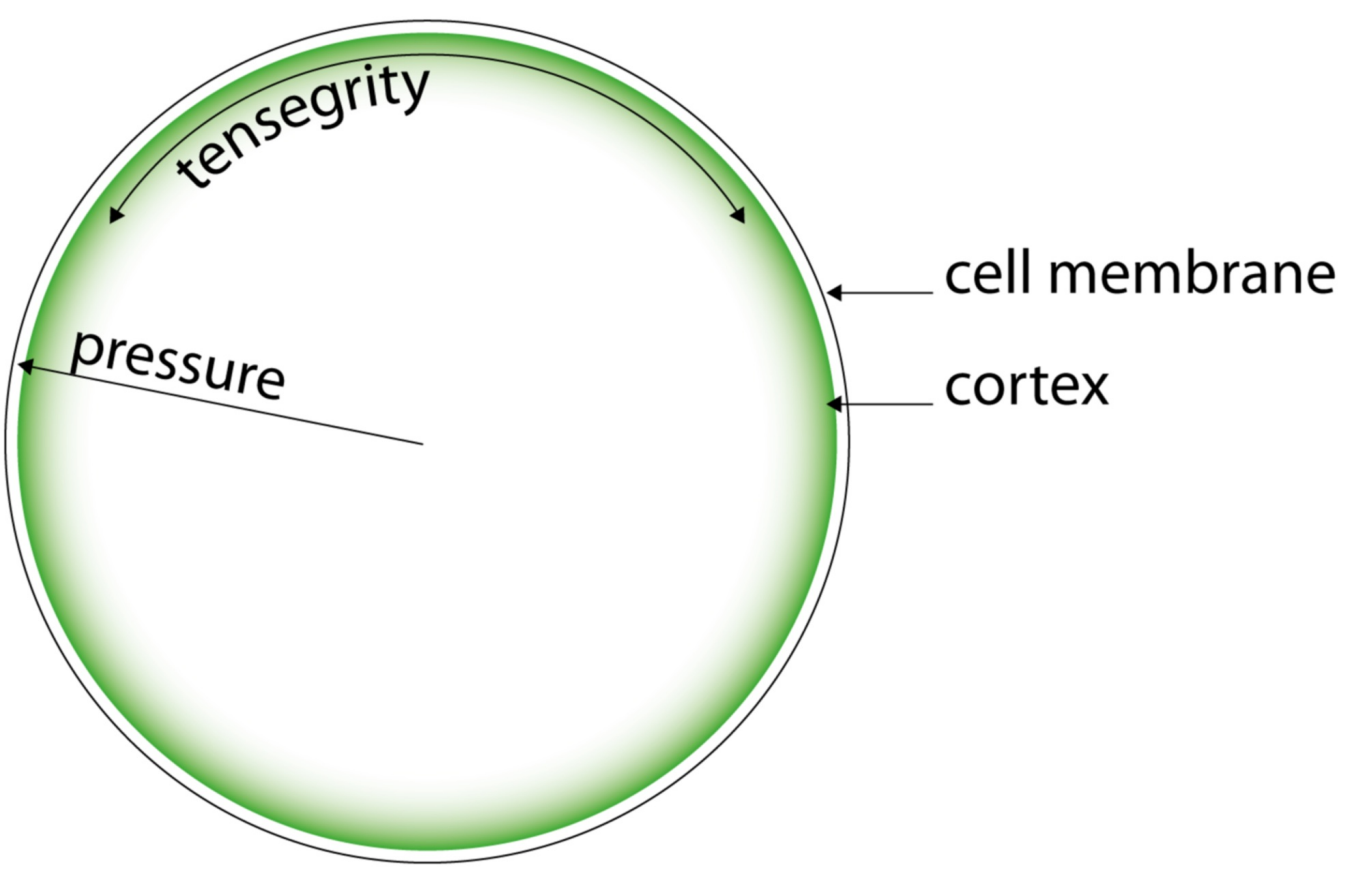
The cellular biotensegrity system

Biotensegrity can be described as a combination of rigid tensional elements. In living tissue, the constituent rigid elements can be small at the molecular level, large as in bones and flexible as seen in soft tissue. However, unlike common mechanical structures, (e.g. the wheel/spokes/rim), tensegrity elements in nature are dynamic, not static but able to adapt. This adaptation, which can occur within seconds or even milliseconds, allows cells and tissues to change shape [12]. A biotensegrity system allows the cell, tissues and body parts to use fluidity to dissipate the energy caused by movement and deformation but still maintain structural integrity, therefore, enabling tissue to handle forces. In nature, combining both types of structural elements (rigid and flexible), cellular pressure and tensegrity allow for harvesting the benefits of both systems whilst eliminating the drawbacks [13]. For example, in the cell, the cytoskeleton is operating together with internal pressure to maintain structural integrity with minimal energy expenditure and without compromising its flexibility and adaptability: achieving tensional homeostasis [14]. In this article, we use the term “biotensegrity” for this combined system of internal fluid pressure: cellular pressure and tensegrity (Figure 4).

**Figure 5:**
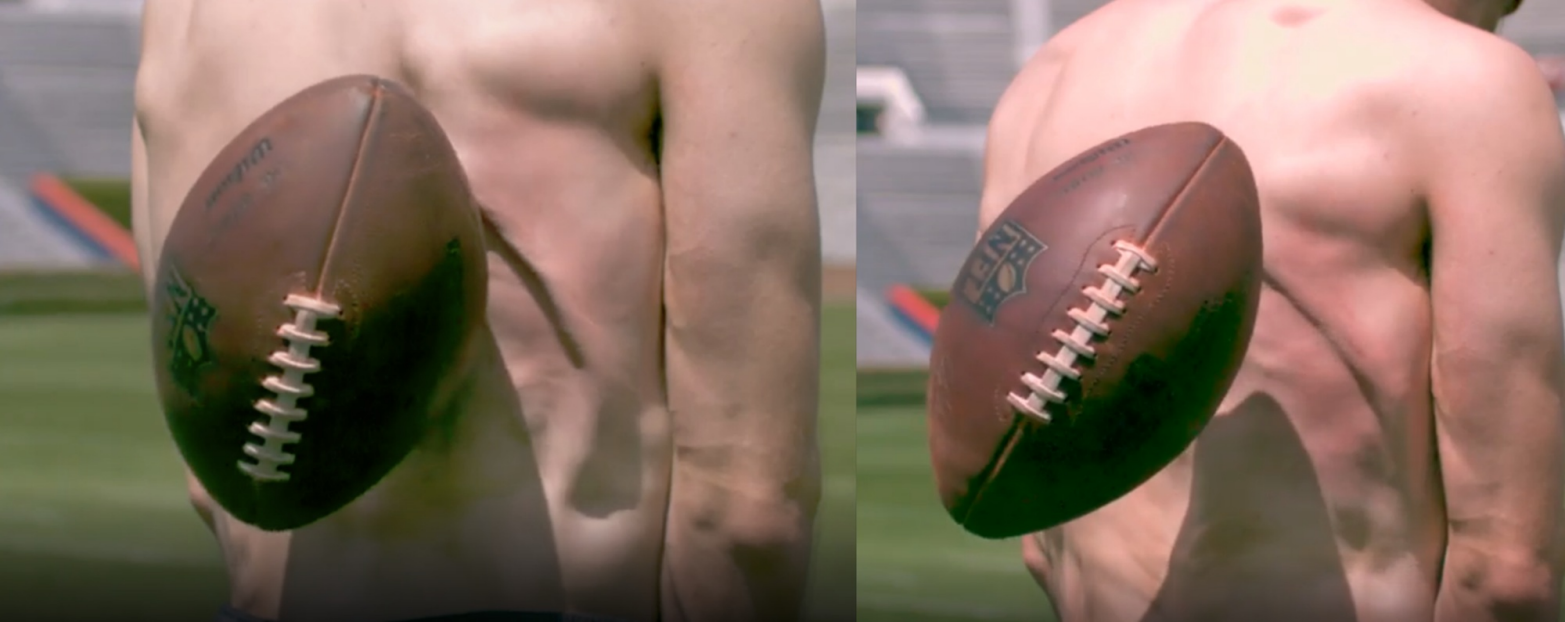
Fluid-like phenomena in human tissue Two sequential pictures where you see waves propagating from the impact area

The same applies to tissue where its combined effect can be observed. For example, in Figure 5, the tissue can be observed to behave as a fluid, yet the movement of the constituent cells is limited and protected by the tensegrity system, which allows for energy displacement without damaging tissue displacement - the energy dissipates in a wave-like manner.

Biotensegrity of the cell

Cells must maintain their tensional homeostasis in order to function optimally and to achieve this, cells use internal pressure and the tensegrity elements of the cytoskeleton [15]. The cytoskeleton has three main components: F-actin, microtubules and intermediate filaments. Intermediate filaments (e.g. vimentins) form a network from the nucleus right into the ECM [16]

**Figure 6:**
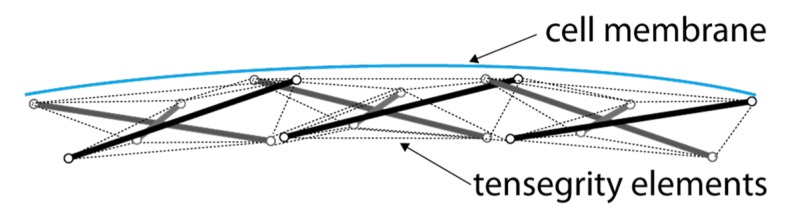
Schematic representation of the cortex

The cornerstone of a cell’s outer strength is the part of its cytoskeleton situated right beneath the cell membrane: the cortex [17]. As shown in Figure 6, the cortex consists of a sub-membrane network of actin filaments combined with myosin II filaments. An actin filament can be stressed by a myosin-II filament resulting in a “prestressed” stronger actin-myosin filament. Prestressed actin-myosin fibres interconnect in the cortex and provide structural support to the overlying cell membrane, not unlike the structures on the inside of a dome [18].

In the context of biotensegrity elements, the cellular cortex are analogous to the “rim of a wheel” where the rest of the cytoskeleton forms the “spokes”. Further strength is achieved through internal pressure or cellular pressure. The combined biotensegrity system harvests the benefits of both systems at minimal energy expenditure.

By repositioning or differentially stressing specific elements within the cytoskeleton, biotensegrity allows both individual cells and tissues to change from a solid to a fluid-like state [19-20]. This change of rigidity can happen within milliseconds and is well-characterised by the events in a muscle, where force is generated by actin:myosin interaction.

Changes in the cortex-associated cytoskeleton elements can, therefore, adapt the rigidity of the cortex rapidly. However, in addition to the cytoskeleton elements of the cortex, further stress can be applied by fibres in the cytosol, which adds extra stress and, in turn, rigidity to the attached part of the cortex under the membrane [21]. Therefore, a cell can change its rigidity by stressing the fibre layer in the cortex internally and by tensioning the cytoskeleton fibres from within the cell [22]. This allows the cell to meticulously adapt the rigidness of parts of the cell wall, allowing for deformation, a prerequisite for movement [23-24]. This adaptability is different from plain rigidity or stiffness [24-25].

The internal pressure of a cell is not only partly maintained by osmotic processes, which are controlled by active pumping of ions through the cell membrane but also the contraction of the cytoskeleton system is one of the ways cells can rapidly change their internal pressure [18,26]. This is how cells such as fibroblasts adapt their tensional state in order to deform, move and respond to slow and rapid deformations resulting from injury [27-28]. Tensional homeostasis is just one of the roles of the cytoskeleton, which has many other functions [29-30].

In summary, the biotensegrity of a cell is a result of the combination of the tensional elements of the cellular tensegrity system, such as the cytoskeleton and fibres, and the internal osmotic pressures. This means that a cell’s structural strength and shape is based upon a combination of cellular pressure and a combined actin-myosin activity. This allows the cell to resist distortion as a whole without compromising its capacity to actively change shape: biotensegrity [31].

Biotensegrity of tissue

Unlike a non-biological Buckminster Fuller structure, in biology, most structures, whether sub-cellular, cellular or larger structures have multiple functions. For example, when producing hormones or producing movement is a primary function, structural integrity is often a minor function. Tensegrity elements in living tissue will, therefore, not always have the shape of a tetrahedral structure. They may have various forms and shapes.

Furthering the intracellular discussion of tensegrity above, in tissues, cells are interconnected through tensegrity elements that extend beyond the cell. For instance, epidermal cells are tightly bound together through cell-cell contacts.

**Figure 7:**
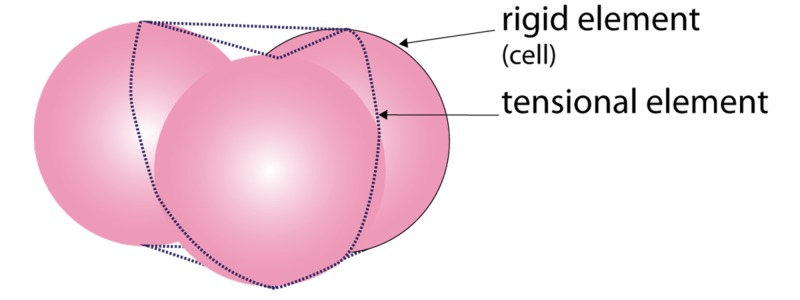
Schematic representation of a tensegrity element at cell scale

The dermis consists of a dense extracellular network [32]. Cell-matrix interaction is achieved through transmembrane proteins called cadherins, which connect the extracellular matrix (ECM) to actin and the intermediate filaments inside the cell [33-34]. This is how the tensegrity elements inside the cell are connected to the ECM and other structural elements. This transmembrane connection leads to the emergence of a tensegrity element of a higher order of scale. At this larger scale, cells have the function of the rigid elements and the connective elements (ECM) are found outside the cell (Figure 7). This system of tensegrity elements consisting of smaller tensegrity structures interconnecting and thus forming larger elements is how the biotensegrity system functions as a continuous force-handling system; a larger element consists of functioning small elements. The function of force handling is similar but the biotensegrity structure can be very different and even appear irregular across differentiated tissue types.

The cell-cell connections are not only structural elements but also provide another means for individual cells and groups of cells to communicate and regulate their behaviour [35]. This connection enables forces to be dissipated between cells by changing their position or fluidity and to share or dissipate any force that could otherwise damage an individual cell. This paracrine communication is necessary for the continuity of the sensory system, which, like the biotensegrity system, must work seamlessly from the level of a single nucleus to the entire body.

The ECM provides both structural support for cells and an extra force-transmitting structure, allowing for disseminating force not only via cell-cell contacts but also via cell-ECM and ECM-ECM contact.

The ECM consists of proteins, glycosaminoglycans and fluids. Due to its biochemical aspects, the ECM also plays an important role in the physiology. The fibrous components of the extracellular matrix consist of three types of fibres: reticular fibres, collagen fibres and elastin fibres [36]. A major constituent of the ECM is the ground substance that has a thixotropic, hydrophilic nature, which allows tissue to change its viscoelasticity when mechanical force is applied [37]. When under strain, the ground substance becomes more fluid, reducing friction between structures and enabling displacement between cells within the associated tissue. It also changes the force-handling strategy from local to more remote or dissipated since displacement allows for the transferring of forces over larger distances. As a result, specific responses of biological networks to forces applied are complex [38].

Collagen type 1 is the most abundant structural component of the ECM, and it has a specific time-related response to mechanical forces [39]. Collagen fibres can handle high tensional loads, which allows for the absorption and dissipation of forces in and between tissues. The latter allows for an increase in the force-disseminating capacity of cells within the tissue, allowing groups of cells to handle damaging forces whilst keeping the forces at the individual cell level within homeostatic boundaries [24]. Not only does collagen allow for this response to mechanical forces, but it can also adapt to continuous forces over time through rather simple mechanisms. Collagen is continuously produced and degraded but the degradation rate of collagen depends upon the tension of that fibre. Tension reduces the degradation of a collagen fibre [40]. Since tension has no effect on the speed of assembly of collagen, over time, collagen fibres will accumulate in the direction of the applied tension, effectively increasing the force-handling capacity in that specific direction and is the driving force of anisotropy of living tissue where the force-handling capacity in a tissue is not the same in all directions. The process of tissue restructuring as a result of the applied force is relatively slow and this means that tissue can adapt over time if the forces are not larger than the tissue threshold. In a longer time scale, collagen fibres and other connective structures in tissue organise themselves through adaptive remodelling upon mechanical forces [41].

The system by which tissue adapts to force is automatic and, therefore, its mechanical and chemical structure will cause the tissue to respond to forces in a very specific way. As a result, there is a reciprocal interaction between the connective elements and cells in a tissue, both respond in their own way to external forces and each other [42]. Essentially, cells and ECM function together to manage the dissemination of force at the tissue level.

A very clear example of the function of the biotensegrity system in tissue is present in adipose tissue. Under compression, the incoming force is not only handled by increasing the internal pressure in the cells but, additionally, the ECM and other larger fibrous elements transfer force through their connection to the fascia. This involvement of the larger structure allows for the dissemination of the force over a much larger volume. Counterintuitively, increasing tension on extracellular structural elements dilates blood and lymph vessels. This differs from the force causing perfusion problems in other tissue types [43]. These complex and sometimes counterintuitive responses allow for handling forces, which would not be sustainable without the complex interaction between intra-and extracellular structural elements. This also leads to the observation that adipose tissue, which appears unharmed may be, in fact, compromised and cause undermining in force-related tissue damage [44-45].

In other tissues like the skin, the size of the structural elements gradually increases the further away they are from the epidermis. This allows for an appropriate response to small and large forces.

Generally, tensional homeostasis in tissue is maintained through internal pressure in and around its constituent cells. This can be observed in dermal incisions where the adipose tissue bulges into the lesion.

Biotensegrity of the body

The basic building blocks for tensional homeostasis, cellular pressure, and tensegrity remain the same throughout all tissues from micro to macro [14,46].

**Figure 8:**
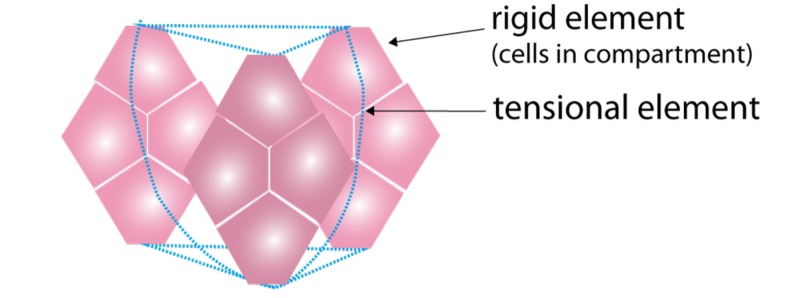
Representation of tissue tensegrity

The larger anatomical structures allow for force transmission over larger distances and/or areas. If all cells and tissues in the entire body were directly connected the same way, tissue would be relatively stiff and hard to bend. This would be problematic, for example, in larger organs or muscles. Instead, the system must allow tissues and cells to change shape and move relative to each other. This is achieved by compartmentalisation, where tissue is divided into smaller compartments and lobes, which are able to move relative to each other as seen in adipose tissue and muscles [47]. Here, tissue compartments emerge as the rigid part in the tensegrity system at the tissue level such as that illustrated in Figure 8.

With the increasing scale of the force-handling elements, the involved structural elements also increase in size. At the level of larger tissues functioning as a rigid element like muscles, bones and organs, the tensional elements also increase in size. At the macro level, this is the fascia.

Discussion 

To avoid incoming forces causing harm, the system must ensure the tissue thresholds to handle pressures are not surpassed. The biotensegrity system, therefore, functions as a robust continuum across the entire body.

Not all tissues, for example, the kidney, have a primary biomechanical function in the body. At a higher organisational level, specific properties will emerge to protect relatively fragile structures and tissues. As such, the force-handling system in these cells and tissues depends on larger structural elements to absorb and distribute forces, protecting delicate tissues like the skull protects the brain from the direct force - rather than the subtype of tissues themselves. This also extends to the protection of such vulnerable tissues against the forces generated by other structures, such as fascia, muscles, tendons and bones, applying unnecessary forces to tissues.

Tensegrity acts across all these systems: from rigid molecular elements, such as actin and the tensional myosin molecules, and through the larger scales of size, where cells function as rigid elements and the connective tissues function as the tensional elements, and one organisational step up where compartments as seen in septae-like structures, play a role in the biotensegrity system. Then, on a higher organisational level, the collective tissues become the rigid element and the connective tissue then forms the tensional element. Finally, tissues together with bone form the rigid element, with the fascia and tendons as the tensional element. Larger elements can adapt through deformation where they exhibit liquid-like behaviour, however, deformation can cause damage. Therefore, if extensive deformation is required, displacement of the constitutive structural elements relative to each other can occur. This is achieved through sliding connective tissue elements. Thus, the body exhibits the ability to deform and to slide allowing biotensegrity elements to transfer forces away from fragile elements towards more rigid elements.

**Figure 9:**
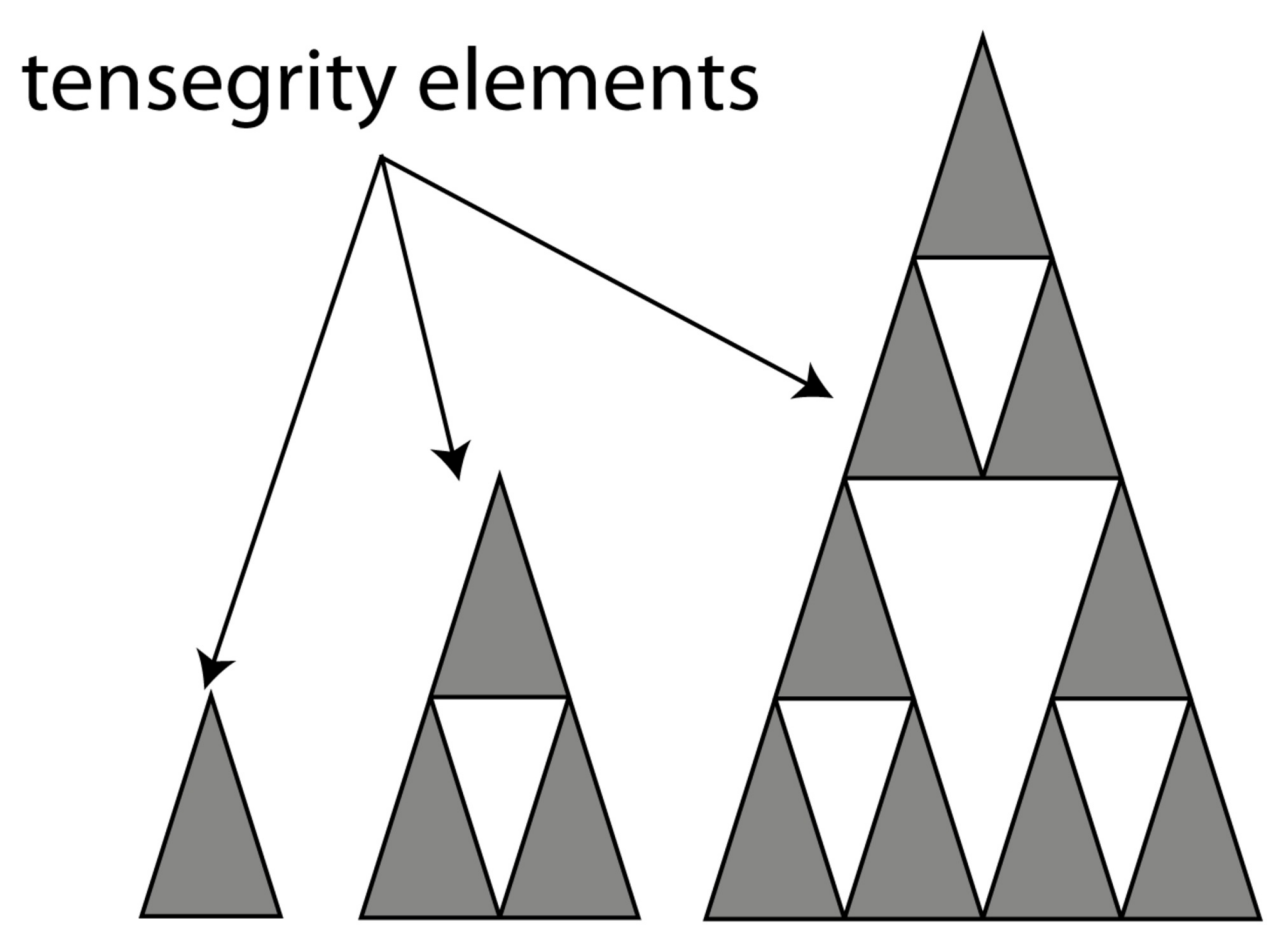
How tensegrity elements maintain function whilst jumping scale

This biomechanical system is not a singular system but an interconnected system consisting of self-stabilising biotensegrity structures at all organisational levels. Interestingly, the rigid elements are non-continuous; they “jump-scale” from one cell, to a group of cells, to tissue, to the entire body, as suggested in the illustration in Figure 9.

The continuity is maintained by the tensional elements; the connective tissue, depending upon the scale, referred to as the cytoskeleton, extracellular matrix and/or fascia. The continual combination of rigid and tensional elements functions as one robust system. Any break in this system may be pathological. Depending on the function and structure of the tissue, the biomechanical properties and its adaptive capacity will differ. They all consist of compartments of varying rigidity combined with filamentous structures. This system allows for adaptation through the movement of elements and by varying the rigidity of cells and tissues. The totality of these features is how biological structures can be rigid but also behave like a fluid, depending on the circumstances and function. This happens not only during the movement of a single cell, but it is also observable in the thixotropic behaviour of tissues that enable our ability to move [48].

To achieve a “biotensegrity continuum” over all the different elements of the biomechanical system, it has a fractal-like design, where the same functional principle is maintained over the many different structures in the body. It spans all scales of the organisational levels, from the level of inside the cell up to the entire body. Only a continuous, fractal-like system can handle forces seamlessly over so many elements.

Implications of the biotensegrity system

The biotensegrity system normally functions flawlessly. In compromised patients, the structural continuity or the functional robustness of the system may be reduced, leading to decubitus and other tissue-damaging events. This implies that apart from visual and (bio)chemical analysis, we can examine our subject through the lens of the biotensegrity system. Therefore, the phenomena of force-related tissue harm; which can develop into a rapid uncoupling of tensegrity elements can lead to deep tissue injury, undermining of wounds, Morel-Lavallee lesions, etc. Biotensegrity may offer useful opportunities for reappraisal in the aetiology and treatment of such phenomena.

Age-related changes and/or comorbidity can cause structural or functional problems that reduce the body’s ability to maintain its tensional homeostasis, leading to harm at points where the continuum is compromised or disrupted. Causes of harm can be straight forward such as immobility. However, the severity of the resulting problems can be increased by issues at the (epi)genetic level, (cell) metabolic level, compromised organ systems (such as neurologic or immunological disorders), reperfusion injury and other physiologic and/or anatomic phenomena that reduce the body’s capacity of handling both forces and preventing the harm resulting from force [49-50].

## Conclusions

The concept of biotensegrity, tensegrity in living systems, provides a deeper understanding of soft tissue biomechanics. A closer look at how nature has organised tissue integrity by means of rigid and tensional elements reveals a system that spans all the organisational levels from the inside cell up to the entire body in a way that does not violate the rules of minimal energy expenditure. This article describes how the biotensegrity system functions in a continuous way by means of interconnected elements of the micro and macro anatomy. Incorporating biotensegrity in the observation of force transmission in and between structures in the body also suggests that the current approach may overlook events and damage, which will have a detrimental effect on the outcome. We have to consider a further systems approach to analyse the effects forces have on the body, in a context that is not static but dynamic and responsive.
